# A precision immuno-oncology turn? Hybridizing cancer genomics and immunotherapy through neoantigens-based adoptive cell therapies

**DOI:** 10.1177/03063127241303720

**Published:** 2024-12-15

**Authors:** Luca Chiapperino, Nils Graber, Francesco Panese

**Affiliations:** University of Lausanne, Lausanne, Switzerland

**Keywords:** cancer immunotherapy, genomics, sociotechnical regimes, hybridization, neoantigen

## Abstract

This article explores the development of T cell-based therapies in Switzerland. These therapies, which elicit the immunological potential of each patient to respond to tumor development, constitute a major promise for so-called ‘precision oncology’. We document how immunological concepts, technologies, and practices are articulated given the centrality of genomics in ‘precision oncology’. We consider ‘precision immunotherapies’ to probe whether and how change ensues in these established sociotechnical regimes of biomedicine. The case of genomics and immunology in oncology offers a unique insight into the conditions of possibility for change in such regimes. How does the present new wave of cancer immunotherapies challenge, integrate, and complement the centrality of genomics in ‘precision oncology’? What are the specific processes that make possible the convergence, competition, or co-existence of distinct conceptions, infrastructures, and programs of innovative cancer medicine? Drawing from observations and interviews with researchers and clinicians, we qualify these sociotechnical processes as *hybridizations.* Bringing together different sociotechnical regimes of biomedical research is conditional to the articulation of core concepts, technologies, and translational practices of genomics and immunology. Pivotal to this objective are neoantigens, cell surface proteins originating from the somatic genetic mutations of tumors and which activate a patient’s immune response. While neoantigens are an unstable entity in experimentation, they offer a conceptual and material substrate to renegotiate the dominance of cancer genomics, and initiate the production of a new, hybrid regime of ‘immunogenomic precision’ in oncology.

Over the past three decades, the landscape of cancer biomedicine has been dramatically shaped by the promises and developments related to ‘precision oncology’. Since the 1990s, cancer genetics—and later genomics—have developed following the idea that genetic mutations (i.e., changes in the DNA sequence of somatic cells) are the main biological drivers of tumorigenesis. Propelled by a growing availability of tools to investigate each tumor’s mutational pattern, cancer genetics/genomics has brought genetic mutations to the fore as ‘hallmarks’ of cancer aetiology and progression (Hanahan & Weinberg, 2000, 2011). The relevance of genetic signatures for cancer extends into a specific therapeutic program of ‘precision’ oncology, which uses specific drugs to act on the molecular machinery of cancer progression driven by mutations. A key feature of so-called ‘targeted precision therapies’ is interpreting and prioritizing mutations identified by genome sequencing in a way that is both complementary to traditional histology-based cancer treatment, and informative of potential targets of molecular therapies (Hahn & Martin, 2015).

Since the 2010s, oncology has also become increasingly bound to the development of immunotherapies. Considered by *Science* as the ‘breakthrough of the year’ in 2013 (Couzin-Frankel, 2013), immunotherapies gained even more importance in 2018 when immuno-oncologists James Allison and Tasuku Honjo were awarded a Nobel Prize for their discovery of immune-checkpoint inhibitors. The targeting of these inhibitors works under a different view of tumorigenesis and cancer development. These therapies target the ‘brakes’ that tumor cells put on the immune system to prevent a patient’s own defensive response against it. Treatments targeting immune-checkpoint inhibitors thus aim at unleashing the production of T cells (CD8), frequently called ‘killer T cells’. More recently, other types of cancer immunotherapies have attracted consistent research efforts, such as adoptive cell therapies (ACT) relying on expanded or engineered tumor-infiltrating lymphocytes (TIL)—T cells found in the patient’s tumor that can ‘recognize’ it and attack it—or vaccination through dendritic cells, a specific population of cells able to present tumor antigens to the adaptive immune system (see Tanyi et al., 2018). Cancer immunotherapies currently populate the promissory landscape of ‘precision medicine’. Along the established emphasis on genomics for ‘precision medicine’, one can record a growing association of labels like ‘precision’ or ‘personalized’ with immuno-oncology (Rosenberg & Restifo, 2015). At the time of writing, some ‘precision immunotherapies’ have already obtained regulatory approval, particularly immune-checkpoint inhibitors and chimeric antigen receptor T cell (CAR-T) therapies—a form of ACT relying on a genetically modified cellular receptor (Adhikary et al., 2024).

In this article, we investigate these immunological concepts, technologies, and practices in so-called ‘precision oncology’. Our aim is to explore whether and how change ensues in the established sociotechnical regimes of biomedical innovation and inquire into the conditions of possibility of change in the epistemic and organizational dimensions of this regime. How are new ideas, tools, and actors involved with immuno-oncology for the sake of ‘precision medicine’? As we detail below, the place of immunological ideas, approaches, and technoscientific tools within the bundle of epistemic practices labelled ‘precision oncology’ is far from evident. In a field dominated by genome-centered research for decades, the integration of cancer ‘precision immunotherapies’ prompts the emergence of changes that go to the core of contemporary oncology. As we clarify below, we qualify these processes as the hybridization of core concepts, technologies, and practices of different sociotechnical regimes of biomedical research and practice.

From a historical perspective, a clarification is in order about the relationship between genomics and cancer immunotherapies in ‘precision oncology’. ACT could in fact be considered nothing more than a new wave of cancer immunotherapy, extending a longer history of these ideas and research practices in oncology. As Löwy (1996) extensively argues, cancer immunotherapy pre-dates the epistemic dominance of genomics in oncology and has developed through ‘waves of enthusiasm’ followed by periods of marginalization. Her study of these therapies—including ACT—in the 1980s argued that cytotoxic T cells constituted a prolific boundary object connecting different fields of research practice around the therapeutic potential of the body’s immune agents. In her analysis, ACT did not however give form to stabilized knowledge or a lasting clinical approach. In fact, in the 1990s—a decade that, incidentally, marks also the rise of cancer genetics (Nelson et al., 2013, 2014)—the field of cancer immunotherapy became largely marginal. In the absence of major clinical breakthroughs, research partly withdrew from this domain, as well as industrial investments. With the current new wave of cancer immunotherapy since the 2010s, T cells re-emerge as central biological agents of cancer therapy, to be combined with immune-checkpoint inhibitors. And they do so in a landscape heavily shaped by cancer genomics.

If cancer immunotherapy has made a comeback after decades of marginalization across the 1990s and 2000s, things stand differently with developments in genetics and genomics. As shown by Nelson et al. (2013, 2014), these last few decades have witnessed the emergence of what these authors have called a ‘distinctive sociotechnical regime’ in oncology; that is, a systematic social and technical effort to integrate genomic ‘technologies with existing treatment routines, clinical trials, regulation, and health care infrastructures’ (Nelson et al., 2013, p. 407). The centrality of genomics—its concepts, tools and practices—as Löwy (2022) has recently argued, can be qualified as that of a ‘scientific ideology’ (p. 487). Genomics is at once a material, conceptual, and social arrangement of biomedicine structuring biomedical research and practice, inspiring and coordinating expert work, guiding the development of promissory therapies, and configuring novel communities of practice. This approach to ‘precision’ has also—although only partially—crystallized as an actionable repertoire of experimental care (Cambrosio et al., 2018). While changing the organization of clinical and research work, genomic ‘precision medicine’ has led to limited results in terms of clinical efficacy (Prasad, 2016). And yet, genomics today structures the theoretical, technological, and practical conditions of possibility of innovation in oncology. Philosophy of science scholarship can be mobilized to highlight the theoretical dimensions of the dominant ideology of ‘genomic precision’: Views of the centrality of mutations constitute the so-called clonal conception of cancer, which has provided a largely-uncontested explanation for cancer in the last few decades (Bertolaso, 2011; Marcum, 2005). Beyond theories, however, the experimental systems of genomics heavily structure the scientific gaze and the ordinary practices of contemporary cancer research (Rheinberger, 2010; Rheinberger & Müller-Wille, 2018). Genomics, it has been shown, offers the technoscientific infrastructure that allows clinicians and scientists to describe the heterogeneity of patient conditions and clinical paths based on specific measurable biological characteristics of individual tumors; namely, their genetic mutations (Nelson et al., 2013, 2014). In a nutshell, genomics makes up a regime of ‘precision’ in oncology, in the sense of constituting a cognitive, technical, and normative structure guiding innovation in this field. It entails apprehending cancer according to a given set of biological features, such as genetic mutations; it structures the way these variables are measurable through specific equipment (e.g., genome sequencers); it suggests these signatures define a patient’s clinical characteristics and susceptibility or responsiveness to treatment. Furthermore, the regime of genomic ‘precision’ can be claimed to extend into clinical research and practices, much like the regulatory frameworks and expert jurisdictions in this field (Beaudevin et al., 2019; Bourret & Cambrosio, 2019; Cambrosio et al., 2018; Kohli-Laven et al., 2011; Rabeharisoa & Bourret, 2009). Genomics is in fact at the core of the conceptual, social, and organizational strategies that promote translational research in contemporary oncology: any approach to spearhead the exchanges between scientific evidence constructed in research settings and clinical routines (Crabu, 2016, 2018).

In what follows, we draw from observations and interviews with researchers and clinician-researchers involved in a translational pipeline of ‘precision immuno-oncology’ at a cancer research centre in Switzerland. We show that a renegotiation of these dominant features of genomics is at stake when researchers experiment with possible hybridizations between immunological and genomic approaches to ‘precision oncology’. We focus on work around ACT and investigate the role of neoantigens in this process—the specific proteins on the surface of cancer cells emerging from unique somatic mutations accumulated by cancer cells that selectively sculpt tumor immunity and/or immune response (Schumacher & Schreiber, 2015). Neoantigens sit at the crossroads of genomic and immunological ways of knowing cancer and operate as enabling factors for the hybridization of cancer genomics and immunotherapies. Neoantigens define the immunogenic specificities of patient tumors, while mobilizing the productive experimental systems of genomics. They remain connected to the epistemic horizon of clonal/genetic views of cancer while affirming the relevance of immunological processes in cancer development. We use the term ‘hybridization’ here in ways that complement previous uses in Science and Technology Studies (Löwy, 1994; Rheinberger, 1997). Hybridization, we maintain, is not merely a sociotechnical process merging communities of practice, research and care settings, or the agendas of science and society (Löwy, 1994). Nor do we focus solely on hybridization as the sociotechnical juncture for experimental systems of contiguous research threads which can complexify biological facts (Rheinberger, 1997; see also Chiapperino, 2024). Rather, we take hybridization to underline a multidimensional process. Furthermore, hybridization can underline how reconfigurations of established sociotechnical regimes of (genomic) ‘precision’ in oncology are fundamentally ‘in the making’. Neoantigens escape a clear definition and are far from arbitrating the theoretical, experimental, and translational differences that characterize genomic and immunological styles of practices in cancer research. Briefly put, they are far from defining once-and-for-all a hybrid regime of ‘precision’ in oncology. If anything, neoantigens trigger hybridization as an open-ended process, a kind of work actors must perform to bring together concepts, tools, and clinical approaches that are far from dissolving into one another.

We shed light on three different modalities of hybridization between cancer genomics and immunotherapy, as operated through neoantigens. First, we analyze *theoretical hybridizations*, by showing how the ‘neoantigens’ concept produces a specific understanding of ‘precision’ which articulates mutation-based/clonal theories of carcinogenesis with immunological views (Bertolaso, 2011; Plutynski, 2018; Pradeu, 2019; Rondeau et al., 2019). We show how neoantigens hold together distant views of the biological basis of cancer through a focus on the therapeutic potential of patient’s own biological material (T cells) for both intervening into cancer immunoediting and targeting its specific mutations. Second, we illustrate how hybridization takes place at the level of *experimental practices*. We show how developing neoantigen-based ACTs heavily relies on connecting the tools of genomics (exome sequencing and bio-informatic analysis) with the equipment of immunology to permit an optimal selection and expansion of immune agents specific to patient tumor characteristics. Third, we illustrate how this theoretical and experimental work marks the beginning of a *translational hybridization* through an epistemic and regulatory reconfiguration of clinical research for ‘precision immunotherapies’. Although we do not directly analyze clinical research practices, nor the therapeutic efficacy of these therapies, we show that the translational research practices connected to neoantigens initiate the integration of genomic and immunological clinical and biological research spaces. While immunotherapies targeting neoantigens are currently a shared notion among researchers and clinicians, they are not necessarily converging into common biomedical platforms (Keating & Cambrosio, 2003). Neoantigens, we argue, are an unstable biomedical entity: as concept, object of experimentation, and actionable biomarker. Yet, they offer researchers the opportunity to connect (*hybridize*) competing research and therapeutic strategies within the landscape of so-called ‘precision oncology’.^1^ Our documentation of these hybridization processes contributes to STS analyses of the conditions of possibility for change in the sociotechnical regimes of biomedicine.

## Context of the research and method

Our fieldwork took place in the New Cancer Research Centre (NCRC) in Switzerland. Following global trends in cancer research and a local tradition of immunological research, the NCRC gives central place to translational research for the development of cancer immunotherapies (see also Chiapperino et al., 2021). The NCRC has been conceived as a flagship institution and a landmark for uniting the local and regional cancer research community. It espouses and reproduces several global trends towards precision oncology: a rapprochement between basic and translational research (Cambrosio et al., 2018), the integration of life sciences tools in hybrid experimental and clinical settings (Nelson et al., 2013), the reconfiguration of professional territories in molecular tumor boards to bring these tools into clinical decision (Bergeron et al., 2021; Bourret & Cambrosio, 2019), the reconfiguration of epistemic territories and pipelines of knowledge-production through the architectural and organizational rearrangements of scientific activity (Chiapperino et al., 2021), and the realignment of basic and clinical research to privilege innovations with high translational value (Crabu, 2016, 2018). Of note, the NCRC was also created to strengthen the relations between researchers and practitioners from the fields of immunology, genomics, bioinformatics and clinical sciences.

In the NCRC’s pipeline, two types of autologous therapies are currently being developed: (1) ACTs relying on TILs, which are selected and expanded (yet for the moment not genetically engineered) *ex vivo* in a manufacturing platform meeting ‘Good Manufacturing Practices’ (GMP) standards before being re-infused to the patient, and (2) cancer therapeutic vaccines made from dendritic cells and tumor cells, both extracted from patients. Translational research projects for both therapies share several experimental steps. In both cases, autologous immune cells with the highest specificity for the mutational landscape of patient’s tumor must be identified, to boost the precision and efficacy of immunological treatment. Both therapeutic strategies entail the tailoring of a patient’s immune response to the biological characteristics—and the mutated genetic features, such as neoantigens—of the patient’s tumor.

This article emerges from interviews (n = 16) conducted between 2019 and 2021 with principal investigators and senior researchers in the fields of immunology (4), molecular biology (3), biotechnology/manufacturing (3), bioinformatics (2), oncology (2), and regulatory affairs (2), all involved in the activities of the NCRC. The semi-structured interviews followed a common guide, organized by three main topics: research trajectory of the interviewee, epistemic dimensions related to cancer and precision immunotherapies, and research organization. All recorded, most interviews took place during the COVID-19 pandemics and were performed through videoconferencing software. Interviews were then coded and analyzed by using the textual analysis software Nvivo. To a lesser extent, we also draw from onsite and online observations of the Molecular Tumor Board (MTB) and the follow-up of clinical trials at the university hospital. Such ethnographic material, based on note-taking, permitted us to enrich our data in relation to the hybridization processes taking place in clinical settings (i.e., in the MTB). Our research abided by the local ethical requirements.

## Theoretical hybridization: Bridging cancer immunology and genomics with neoantigens

Cancer immunology and genomics differ in several ways. They stem from distinct traditions, cluster around partially overlapping experimental settings, and are practiced by diverging expert communities in oncology. These differences also play out as theoretical disagreements about how to explain cancer as a disease, which in turn affect the ensuing therapeutic strategies (Plutynski, 2018; Pradeu, 2019). Before detailing their role in integrating genomics and immunology in experimentation (see next section), this section briefly analyzes how the concept of ‘neoantigen’ produces a conceptual linkage between cancer immunology and genomics in the NCRC.

Biomedical scientists define ‘neoantigens’ as the specific immunogenic proteins on the surface of cancer cells, which originate from the unique somatic mutations accumulated by cancer cells. Neoantigens are therefore the latest iteration of a long-standing question in cancer immunology: what are the factors that modulate a patient’s specific immune response to cancer? The emergence of the concept is due to two seminal studies from 1988 and 1996: The first (Plaen et al., 1988) reported anti-tumor T cells’ recognition of protein products from tumor specific mutations. The second (Robbins et al., 1996) confirmed the existence of these patient-specific mutated proteins in melanoma cells isolated from a patient. These findings suggested a plausible mechanism in support of a nascent view of the participation of the immune system in cancer (Jiang et al., 2019). Known as the immunoediting theory proposed by immunologist Robert Schreiber and colleagues (Dunn et al., 2002; for theoretical scrutiny, see Pradeu, 2019), this perspective postulates that the components of the immune system (innate and adaptive) and the tumor *shape each other*. Besides detecting and eliminating altered cancerous cells, neoantigens suggest that tumor variants actively mutate to escape this control, with the immune system and the tumor adapting their response to one another, which often leads to a tumor escaping the host’s control. Immunoediting highlights, in this view, a diachronic and complex view of cancer development. Of note, this process also made a place for immunological views in the second edition of the highly influential review on the ‘hallmarks of cancer’ by Hanahan and Weinberg (2011), which describes the genomic drivers of a tumor’s biological capabilities. Yet, taking it as a ‘still-rudimentary demonstration’ of the immune system’s role in cancer, the authors considered that the value of this process as a hallmark still had ‘to be firmly established’, and they do not mention the role of tumor mutations producing neoantigens in driving this process (p. 661).

Things stand differently a decade later. As mentioned, the field has witnessed the discovery of immune checkpoint inhibitors, which act on the ‘brakes’ the tumor puts on the immune response. Neoantigen-based therapies have gained increasing relevance through a few landmark documentations of patient remission from ACT (reviewed in Jiang et al., 2019). Throughout the 2010s, neoantigens have established themselves as the support for ‘an ideal immunotherapy’: They are the product of the genetic hallmarks of cancer cells ‘distinguished from germline’, and therefore offer a unique therapeutic target, which can be ‘recognized as non-self by the host immune system’ (p. 2). Used in combination with the blockade of immune checkpoints, ACTs promise to simultaneously break the immune tolerance exhibited by tumors and enhance the functionally adaptive and patient-specific response of immune agents toward tumors.

These elements are key to understanding how neoantigens allow NCRC researchers to conceptualize a precision oncology research program that straddles genomic and immunological approaches to cancer. A translational researcher (with a background in immunology) coordinating a lab that develops ACTs explains how generative the theoretical premise of neoantigens is. As a concept, ‘neoantigens’ enable a therapeutic program that, theoretically, simultaneously builds upon and departs from the approaches of immunotherapies and genome-based targeted therapies in isolation. The jargon he employs to postulate this hybridization is not that of precision, but the cognate nomenclature of personalization, which is far more common in Europe (European Science Foundation, 2012):

[The first ACT we developed was already] *personalized* because it’s the autologous tumor, but now comes the fancy definition of *private* neoantigens. … Private, because the likelihood to find the same mutation in two patients is very unlikely, right? These mutations occur stochastically so your genome is three billion base pairs and any of this is mutated … We developed a way to [characterize the impact of mutations on the cancer cells’ antigens] so that at the end you have a therapeutic product … more tumor reactive and theoretically more efficient. (Immunologist, emphasis added)

There are several elements worth commenting on in this statement. First, note that the semantic shift in the use of ‘personalization’ for neoantigen-based ACTs is not neutral. ‘Personalization’—a term that emerged with the development of pharmacogenomics and targeted therapies (Hedgecoe, 2004)—refers here to a sharply different therapeutic objective: fostering an endogenous immune response from the patient through the selective expansion and reinjection of neoantigen-specific autologous T cells into the patient’s body. Rather than pointing to the singular combination of biological/molecular parameters characterizing the disease of a specific individual (Cambrosio et al., 2021), these ACTs shift the focus of personalization to a therapeutic project based on the patient’s own tailored (immunological) response to the disease. As such, these therapies operate in a semantic space of personalization that is different from pharmacogenomics and targeted treatments. Instead, they sit at the crossroads of immunoediting theory and cell-based regenerative therapies (Gardner & Webster, 2017). They follow the tradition of immunoediting, because their tumor-suppressive capabilities depend on the endogenous immune response of the patient and not the tumor itself (Pradeu, 2019). They designate a process of self-defense of the body that is part and parcel with the long-standing language of immunology (Swallow, 2024). Furthermore, ACTs are contiguous with regenerative medicine, in that they aim at restoring the ‘natural’ tendency of the body towards the re-establishment of normal function; ‘personalization’ here consists more in accompanying the body’s intrinsic vital capacity to restore health, than in the administration of artificial remedies to eliminate morbid states. In this respect, they can arguably be regarded as orthogonal to the epistemic space of pharmacogenomics and targeted therapies still based on conventional treatments like drugs and synthesized molecules. They exemplify a conceptual move from the enhancement of the body’s capacity to suppress the tumor through therapeutic foreign agents, towards a process of ‘normalization’ of the body’s response to disease (Pradeu, 2019).

Second, neoantigens in this context produce a juncture for these diverging views of personalization, at least theoretically. While ACTs prompt a semantic shift in the actor’s use of ‘personalization’, neoantigens enable them to inhabit the same epistemic territory as genomics. For the informant above, neoantigens originate from the ‘private’ (i.e. patient-specific) patterns of genetic mutations acquired by a specific patient’s tumor. They result from the DNA mutations defining cancer cells. As such, they reposition ACTs in full continuity with those genetic processes and aberrations that are today considered central to carcinogenesis and progression (Plutynski, 2018). Much like the progressive accumulation of somatic mutations in oncogenes and tumor suppressor genes, neoantigen formation is (yet) another gene-related event that produces cancer. They are just another of the many genetic vulnerabilities that define tumor characteristics and patient trajectories in contemporary oncology. Under this interpretation, neoantigens can therefore be fully associated with genomic approaches to precision and personalization. Yet, as argued above, they also provide a change in perspective to those views of personalization that focus on genetic mutations. Similar to any immunological process of antigen recognition by T cells (e.g. pathogens, viruses), neoantigens support the principle that these mutations are relevant insofar as they are understood as part of the immunological dysregulation of the body. In a nutshell, the excerpt illustrates how neoantigens are a theoretically prolific notion facilitating crosstalk between seemingly distant theoretical paradigms in contemporary cancer research (Bertolaso, 2016). They provide a common ground on which distinct traditions of cancer research can share a specific experimental and therapeutic program towards precision and/or personalization.

Yet, it is worth noting how this theoretical work around neoantigens is far from settled. While inspiring research programs combining many perspectives, neoantigens still retain substantive interpretive flexibility. In fact, neoantigens have an uncertain status, allowing scientists a dual framing of their participation in such research endeavors. As our interviews show, cancer explanations grounded mainly in genetics can still be considered the default view, or ‘the very basics’ of precision oncology. Take as an example the following researcher with training in cancer genomics, but with a line of research on the tumor microenvironment and neoantigens in tumor plasticity. According to this researcher, immunological concepts and frameworks of explanation are unfinished knowledge that leave untouched a core theoretical understanding of cancer as a genetic/clonal disease. When asked the direct question, ‘What is cancer?’, she replied:

I would like to see the answers of my [immunology] colleagues! What I define as cancer is a genetic disease, so I don’t know how much this is biased by my genomic approach [she laughs]. But, for me yes, cancer is a genetic disease, it comes from the genome and it’s a way to evolve really well, so the cells tend to survive to their lives, they want to become immortal. Cancer is something you acquire through mutations. (Molecular biologist)

This interview excerpt shows that the theoretical elaboration of a common therapeutic program of precision and/or personalized oncology enabled by neoantigens does not require reconsidering or merging explanatory models of tumor onset and progression (see also Chiapperino et al., 2023). First, neoantigens notwithstanding, this respondent’s view illustrates how theoretical disagreements persist in the NCRC. She is aware of her declaredly different perspective, emphasizing the causal role of genes, and how that sets her apart from other colleagues with a strong immunological orientation. Second, the validity of her view seems independent from or unaffected by the prominent orientation towards neoantigens and immunological thinking in the NCRC. Notwithstanding her practical engagements with this research, and the hybridities that neoantigens encourage and support, the development of precision immunotherapies remains another pragmatic version of the (dominant) regime of genomic precision grounded in key genetic elements of cancer. Neoantigens demand little theoretical work because they support the view that cancer is a genetic disease.

Also the answers of respondents with a background and far deeper faith in immunological explanations of cancer avoid any theoretical repositioning on the definition and theoretical explanation of cancer. For a senior immunologist working on cutting-edge methods for neoantigen discovery, the concept of neoantigen can be used to do a different and thoroughly complementary kind of theoretical work: carving a space for genomics within an understanding of cancer as a fundamentally immunological and tissue-based disease.

For me cancer is a cellular disease depending on your immune system. It is a disease coming from your own tissues. Yes, DNA damage creates an uncontrolled proliferation of cells. This changes the properties of the cells, they secrete new molecules, etc. … They stop secreting, or producing some molecules. They create barriers, they become invisible to the immune system. It’s a system of interaction of genes for a cell but then *it is a network inside a network*, because genes are simply something at a deeper level that integrates whatever happens at a higher level. (Immunologist; our emphasis)

For some immunologist interviewees, studying genes’ involvement in cancer (as in the case of research on neoantigens) means simply describing some of the molecular forces behind the higher-level process that is host-tumor dynamics. The disruption of tissue architecture through the loss of immune reactivity is the rather prominent, ‘higher-level’ causal event under this view. Although it is possible to describe this process at the level of DNA, genetic alterations matter insofar as they impact on cell capabilities, or have consequences on tumor cell immunogenicity, or influence the complexity and organization of the tissue turning cancerous (see Pradeu, 2019; Rondeau et al., 2019). The DNA mutations that produce neoantigens are thus either a consequence of higher-level disruptions of cell-to-cell and cell-environment interactions, or an epiphenomenon at the level of DNA, with the causes of cancer occurring at the higher levels of cell structure and tissue immunogenicity.

This conceptual work around neoantigens illustrates how demarcations and convergences of epistemic traditions, specialties, and orientations can be central or sidelined within collaborations among experts with heterogeneous theoretical positions. Neoantigens enable the hybridization of dominant understandings of cancer focused on the centrality of genetic aberrations in carcinogenesis with an understanding of the disease as an adaptive process of tissue architecture, cellular microenvironment, and immunoediting. Yet, far from delivering on a unique and stable configuration of theoretical hybridity, neoantigens set in motion hybridities that are generative from scientists’ situated perspectives. Rival explanatory logics in cancer research need *not* converge around this notion to understand univocally cancer development and progression (see also Chiapperino et al., 2023). The potential theoretical tensions and resistances this hybridization might encompass can be relegated to the background. Neoantigens allow scientists enough convergence and interpretive flexibility to join a specific type of translational research, all while preserving their own explicitly diverging theoretical views. These epistemic practices hybridize an analysis of each patient’s tumor genome with an assessment of its impact on immunogenicity. As such, neoantigens are a pragmatic shortcut that raises the question of whether the complexity of cancer is fully understood and properly theorized across rival research traditions and theoretical orientations of cancer research.

## Experimental hybridization: Producing immunogenomics

Another form of hybridization prompted by research on neoantigens consists of connecting genomics and immunology as independent experimental systems of cancer research. This form of hybridization is known to STS analyses of the historical and epistemological dimensions of shifting material substrates of scientific research (Rheinberger, 1997, 2010, Part 1). In this section, we detail how neoantigens do not present themselves primarily as conceptual hybrids-in-the-making to NCRC’s researchers. Rather, within projects related to ACTs, one of the central endeavors of researchers is to connect experimental systems that are originally unconnected to leverage the potential of neoantigens. These novel arrangements allow them to obtain unprecedented results (Rheinberger, 1997), such as neoantigen-based selection of T cells displaying high levels of specificity with a patient’s cancer. As we shall see, experimental hybridizations open novel spaces for cancer precision therapies that bridge genomic technologies, bioinformatics, and immunological *in vitro* methods.

As a first step, scientists at the NCRC must identify neoantigens. With the development of sequencing technologies, we learned, it has become quicker and more practical to identify and quantify neoantigens specific to each patient’s tumor. A clinician-researcher with a background in immunology told us: ‘Back in the 1990s we vaguely knew about the importance of neoantigens, but we lacked the analytic tools to identify them. Now, in the blink of an eye, we get the genome of a cancer.’ In the 1990s, several research teams attempted to identify antigens specific to a patient’s tumor that cytotoxic T cells could recognize (Jager et al., 1998). The method was a laborious and limited one, which relied on mRNA extraction, a cDNA expression library, and serological methods that built on *in vitro* cultures of expressed peptides and cytotoxic lymphocytes (Kandalaft et al., 2020). With the advent of sequencing technologies—in particular, Next Generation Sequencing (NGS)—and mass spectrometry (MS), it has become possible to investigate a large quantity of fragments of proteins (i.e., peptides). NGS and MS not only gave scientists access to biochemical information about the composition of cell surface proteins, but enabled the identification of protein complexes that emerge from the inherent genetic instability of tumors and produce neoantigens recognized by the immune system. In the NCRC, such practices take place within a research infrastructure called ‘antigen discovery’, where specialists from the growing field of immunopeptidomics (Admon & Bassani-Sternberg, 2011) study the repertoire of peptides related to the Major Histocompatibility Complex (MHC), or human leukocyte antigens (HLA) for humans. A biochemist with recognized expertise in immunopeptidomics—and a pivotal role in the local antigen discovery platform— explains to us that the HLA peptides are among the main immunological entities that ‘present’ peptides at the surface of cells, thus permitting the ‘recognition’ of (malignant) cells by T cells through distinguishing them from normal (self) ones. The same interviewee then elaborates that this kind of research draws from knowledge of the tumor-specific mutations linked to HLA to compare them with normal cells of the same patient:

So we have to first perform a genomic analysis of the tumor and the germline sample from the same patient, and find mutations that are patient-specific in the tumor. Then, what we do is either to perform experimental, biochemical, assay to extract those peptides from the tumors and measure them by mass spectrometry. Or, as an alternative, we apply prediction algorithms on genome sequencing data that would predict which of those mutations are likely to be presented as surface proteins of the tumor. …We combine both approaches because they are kind of very much interconnected. (Biochemist)

This approach of immunopeptidomics combines exome sequencing—an NGS-derived analysis detecting the mutations specific to the protein-coding genome of patient’s tumor—with MS, a technology identifying the peptides at the surface of cancer cells. On a practical level, this means that the antigen discovery platform receives the patient’s tumor sample from the university hospital and realizes the exome sequencing at an in-house facility situated at the partner university. Within the walls of the NCRC, MS analysis is then carried out, before *in silico* approaches take over. The immunopeptidomic collaborators perform bioinformatic analysis of the MS data, while a team of computational biologists specializing in NGS runs the algorithms predicting neoantigens (see Jaton, 2023). The combination of these two big data technologies is taken to deliver on their actionability and trustworthiness (see Tempini & Leonelli, 2021). Uncertainties about MS concern the sensitivity of this technology: it can only detect a relatively small fraction of the total amount of peptides represented by MHC molecules on the cell surface (Bassani-Sternberg & Coukos, 2016). This limitation can be mitigated through its combination with NGS and the use of predictive algorithms, which identify a much larger list of peptides considered as candidate neoantigens. However, this latter approach has its own limitations too: it tends to produce an elevated number of false positives since ‘not all amino acids within a given protein are equally accessible for presentation’ (Bassani-Sternberg & Coukos, 2016, p. 13). In other words, the platform of immunopeptidomics relies on an articulation of high-throughput genomic and protein screening, wet lab, and dry lab approaches, which allows these scientists to identify neoantigens through a two-step method usually starting with NGS and followed by MS to ‘refine’ the detection of neoantigens (p. 13).

These synergies notwithstanding, concerns about the reliability of immunopeptidomic methods abound among our respondents, and especially among the immunologists working in a so-called platform of ‘immune monitoring’. In the case of ACT development, immune monitoring aims to identify and select the tumor-specific T cells, before culturing and expanding them to produce the ACT. A senior immunologist who works in this NCRC platform shared his doubts about the prediction of neoantigens through immunopeptidomics alone:

Neoantigens are … you have to prove them, you have to validate; some exist, but everything starts from mass [spectrometry] data or prediction. … My main concern is everything relies on bioinformatic analysis that are currently being developed and we are not sure that we are reaching for the right neoantigens. So, if the prediction is wrong, finally you could end up with a population enriched in neoantigens that you inject into the patient, but that are not the ‘good’ antigens. You are just injecting nothing, because perhaps the tumor could never express them or they have never existed. … (Immunologist)

While the combination of MS- and NGS-based experimental systems constitutes a prolific configuration to experiment with neoantigens through the tools of genomics, this patchwork of experimental systems is far from being a fully integrated and validated setup for the identification of suitable neoantigens. Rather, it generates new tensions and uncertainties, and calls for the establishment of technical routines, as well as implicit and/or explicit standards, which can guarantee the coherence and validity of this configuration. Chiefly, this causes tensions with immunologists, who were used to working without the high throughput of *in silico* experimental systems, and instead selected TILs according to their *in vitro* ability to bind to tumor cells. Reliant on specific equipment, immune monitoring constitutes a pre-existing way of experimenting with both immune cells and tumor antigens in the NCRC. To spot the T cells binding to cultured tumor cells, researchers from the immune monitoring platform usually perform different *in vitro* procedures, such as: enzyme-linked immunosorbent assays (ELISA) enabling the detection of interferon gamma as evidence of reactivity; mass cytometry to characterize the expression of different markers on cells; and Fluorescence-Activated Cell Sorting (FACS) to sort the specific T cells able to bind to tumor antigens (Bobisse et al., 2018). Furthermore, the patient’s cells (TILs) are cultured for the purpose of these validation and sorting activities. Developed in the 1970s and 1980s, this equipment is illustrative of how knowing immune reactions is intimately connected with the tools, materiality, and values of cancer immunology (Cambrosio & Keating, 1992, 1995). Heavily based on culturing cells (Landecker, 2007), these different immuno-assays are now used to reproduce *in vitro* and *ex vivo* (in the case of autologous cells) the dynamics of T-cell-to-neoantigen binding that takes place in the tissue. They probe the functional characteristics of T cells, such as their ‘fitness’ in culture or their affinity and avidity to bind to neoantigens (Bobisse et al., 2018). As such, immunopeptidomics and immune monitoring require completely different material infrastructures for research.

However, at the NCRC, these different domains and their related equipment have been recently placed in a spatially integrated area, with a low level of outsourcing of experimental procedures, and an explicit commitment to enhance convergence and integration (Chiapperino et al., 2021). Another immunologist explains to us that the techniques of immune monitoring are currently being used in the NCRC to probe/validate the immunogenicity of neoantigens predicted through bioinformatics:

So once we have the bioinformatic list of [neoantigen] candidates for each patient which can be 100, 200, less, depending on the tumor then there is the big part of *in vitro* screening in the lab, the T cells, TILs mainly … so in the blood, to check if these are indeed immunogenic or not. And it can really depend on the 100, 200, you can get two hits, you can get one hit, you can get zero hits. … And then once you know yes that type one and five are immunogenic, then you can play around with it, the affinity, are they functional, are they not functional? Can we pick the best one that has you know the highest affinity or produces more interferon gamma. (Immunologist)

In the novel configuration of the NCRC, one can witness the convergence of these infrastructures of immune monitoring and neoantigen discovery based on NGS and MS. On the one hand, immune monitoring—formerly an independent infrastructure for screening and expanding immune cells based on their ability to bind to tumors—plays the role of validating whether neoantigens predicted through MS and NGS are indeed immunogenic (i.e., whether they represent veritable targets for immune agents such as T cells). On the other hand, immunopeptidomics delimits the potential targets of immunogenicity to the few hundred ‘hits’ identified through MS and NGS, thus making the work of laborious *in vitro* and *ex vivo* methods more targeted. This configuration combines the insight gained from cutting-edge bioinformatic prediction of the immunogenic consequences of cancer mutations, and the reliability of long-standing cell culture methods to reproduce this biological process in laboratory conditions. This hybrid experimental configuration is meant to stabilize the neoantigens with therapeutic potential at the crossroads of those predicted by immunopeptidomics and those that can be cultured to display immunogenicity. Specifically, in the face of a number of candidate neoantigens and T cells, this combination of methods aims at singling out the lymphocytes able to bind to cultured tumor cells. From tools originally conceived to enact competing strategies for antigen discovery, immunological and genomic techniques in the NCRC transform into a novel hybrid setup that borrows objectivity from both sides: the uncertain but high-throughput potentialities of statistical genomic predictions and algorithmic biases are made to converge with the cultured but laborious reproduction of bodily immune reactions in a dish.

Weaving an experimental hybridization around neoantigens is a way to solve the tensions generated by these experimental systems when converged in the NCRC. The experimental work around neoantigens therefore cannot be read simply as a new hierarchy between competing traditions and infrastructures of research. The dominant regime of genomics does not replace long-standing immunology practices of culturing cells through immunopeptidomics, nor is the latter reduced to an ancillary function by the validation role of immune monitoring techniques. the tensions arising at this crossroads are generative and give rise to a hybrid (epistemic) formation: We witnessed neither the replacement of *in vitro* and *ex vivo* techniques of immunology by NGS and *in silico* methods, nor resistance by the former to integrating the latter. Rather, the articulation, merging, and connection of *a priori* separated experimental settings is a creative and productive endeavor (Rheinberger, 1997). Immunopeptidomics—with its reliance on MS, algorithms, and genomics—enhances its reliability and accountability thanks to the validatory step of immune monitoring techniques. The laborious culturing of immunological reactivity benefits instead from the high-throughput discovery afforded by immunopeptidomics. This is not to say that the limitations intrinsic to each research program disappear through hybridization. As we have underlined throughout, the hybridization processes we studied are just emerging: Several challenges lie ahead for fine-tuning an immunogenomic understanding of the cellular specificity and reactivity of a patient’s own immune response to cancer. Yet, the result of this experimental hybridization is the production of unexpected qualities in the NCRC experimental infrastructure. This merger can help develop more personalized ACTs, by leveraging the throughput and rapidity of immunopeptideomics, as well as the reliability of immune monitoring, in the manufacturing of the treatment (we will discuss this further in the next section). Even if, as one of our respondents argued above, it is actually far from clear whether such an experimental hybrid will be able to effectively leverage neoantigens, this complex hybrid formation of experimental systems (cf. Rheinberger, 1997) succeeds in stabilizing them as epistemic objects of research in the NCRC. Beyond any theoretical debate, dispute, or unfinished hybridization, this is made possible at the intersection of the material/technological infrastructures of distinct traditions of cancer research.

## Translational hybridization: Aligning platforms through a ‘pipeline’ of precision immuno-oncology

A third type of hybridization in the NCRC relates to ‘translational research’, a term widely used within the biomedical sciences to refer to the processes and policies aiming at transforming basic research outcomes into clinical innovations. More than just a process going back-and-forth from the clinical to the laboratory settings, translational research in the STS literature is a multidimensional and multimodal dynamic of interaction between clinical and biological research spaces (Crabu, 2016). In the case of cancer genomics, translational research is characterized by the standardization of molecular tests, biological and clinical research focused on actionable pathways (Nelson et al., 2013, 2014), and clinical decision-making practices within Molecular Tumor Boards (MTB; Bourret & Cambrosio, 2019). This specific organization of the back-and-forth workflow between biological and clinical settings has led to a ‘fading boundary’ between research and care practices (Cambrosio et al., 2018). This section details how the clinical development of neoantigen-based ACT at the NCRC shapes a distinct configuration of translational research at the crossroads of two pre-existing types of bio-clinical research infrastructures. One consists of the genomic platforms configured around sequencing technologies to identify mutations, which connect to clinical decision spaces (the MTBs). The other consists of the pre-existing infrastructures specific to ACT, which includes an in-house manufacturing facility involving small bioreactors (in the case of ACT, for the *ex vivo* expansion of TILs found through biopsy) and a dedicated hospitalization unit for the administration of these immunotherapies. While actors in the NCRC invest neoantigen-specific ACT with the potential to hybridize these two existing infrastructures, below we illustrate the multiple ways they are still partially disconnected from one another.

At the time of our research, a pioneering phase I clinical trial involving neoantigens-based TIL-ACT was about to start. Previous clinical research on TIL-ACT did not select T cells according to neoantigens but consisted of the isolation of the whole population of TILs found in the tumor’s biopsy, its culturing and expansion *ex vivo*, and its re-infusion into the patient’s body (a procedure known as ‘TIL therapy’). The new clinical trial aims to leverage the specificity of neoantigen recognition to make this process more effective. If the reinfusion could be restricted only to a T cell population enriched for TILs with a high match and specificity to the tumor’s mutational landscape, then the therapeutic benefits of ACT would be ‘more precise’ and/or optimized. This translational configuration raises an issue of clearance: It is the first clinical application of neoantigen-specific ACT. The leading immunologist who elaborated on the role of neoantigen-based ACT for personalized medicine expressed enthusiasm about this trial. Yet, he noted the complicated work required to meet the regulatory requirements:

So, this is the trial that we [are starting now] … So, we had to GMP-ify the entire process. So, with Swissmedic, it is extremely stringent and painful, it took time, but now it has been submitted to Swissmedic and we hope to start the trial later this year. It is like TIL therapy but on steroid if you want; the maximum you could get out of the TIL therapy. (Immunologist)

In Switzerland, as in most countries, the production of ACT for clinical use must meet Good Manufacturing Practices (GMP), a global standard for ensuring quality and safety followed by most drug regulatory agencies—Swissmedic, in this case. The emic category of ‘GMP-ification’ has complex regulatory, organizational, and experimental ramifications (see also Lewis & Atkinson, 2011). In the phase I protocol, GMP-ification required tailoring the experimental procedures for neoantigen identification and validation to the standards of the manufacturing facility approved for clinical use. Thus, the NCRC researchers, with the assistance of an internal regulatory affairs team, had to produce the regulatory knowledge to convince Swissmedic of the safety of injecting patients with TILs, of the viability of the procedure for clinical use, as well as of the reliability of their selection based on the TILs’ ability to bind to predicted neoantigens.

The production of such a specific ‘regulatory objectivity’ (Cambrosio et al., 2006) can be fruitfully interrogated as another form of hybridization. As intended by Cambrosio et al., ‘regulatory objectivity’ points to the multiple ways innovations are made stable through specific regulatory work that renders them compatible with the knowledge, organization, and standards proper to a wider assemblage of biomedical infrastructures of research and care. In our case, GMP-ification entails producing the procedural standards of the hybrid experimental system connecting immunopeptidomics to immune monitoring. This entails making bioinformatic algorithms of neoantigen prediction travel across borders of basic life sciences research. This means making sure that each experimental step in the antigen discovery process uses reagents, technologies, and methods that are compatible with clinical research standards already in place at the manufacturing facility for ACT. In this respect, GMP-ification is therefore a process expanding the interactions between clinical and biological research settings. It allows the alignment of research activities behind the need to meet procedural and therapeutic standards, setting the conditions for integration within a clinical trial. If a given technical breakthrough is ‘not GMP-ifiable’, one of our respondents asked rhetorically, ‘what good is it?’ (Immunologist). On a broader level, GMP-ification also hybridizes the practices of professionals belonging to distinct genomic and immunological biomedical infrastructures of translational research, if not their distinct expert cultures spanning over basic and clinical research. As we argued elsewhere (Chiapperino et al., 2021), the NCRC is organized around a translational ‘pipeline’, which in this context is ‘both a disruptor and an operator of the alignment of the physical, practical and epistemic organization of these different experimental systems’ (p. 35). Typical of translational research jargon, the term ‘pipeline’ entails a certain sense of verticality and organizational rigidity requiring local biological and clinician researchers to align with norms of accountability and a common vision crafted by the research organization (Lewis & Atkinson, 2011). The GMP-ification of ACT is another catalyser of this process, hybridizing existing platforms in ways that redefine practical standards, research practices, and access priorities into translational and, ultimately, clinical spaces. Still in need of elaboration in our analysis is how this translational hybridization combines the ways cancer immunology and genomics currently span over research and care settings.

In fact, neoantigens also prompt mundane and informal translational hybridizations, all while entering the clinical realities of genomics and immunotherapy. As an example, during our fieldwork, neoantigens were being considered within the clinical decision setting of the MTB. MTBs are part and parcel of the biomedical platforms typical of the genomic turn, since they involve a collective of specialists deciding treatments based on an ‘interpretation’ of variants (genomic alterations) stemming from sequencing technologies (Bourret & Cambrosio, 2019). Indeed, in this clinical decision-making space, we witnessed the notion of ‘tumor mutation burden’ (TMB) increasingly considered in relation to neoantigens. While a leading clinician-researcher considers the notion of TMB too flexible or even poorly defined, it also constitutes another way to potentially produce the hybridization of genomics and immunology in the clinical gaze of the MTB:

The definition of TMB is the number of non-synonymous somatic mutations per megabase, so we try to really quantify the number of mutations by unit of length of the DNA, and the cut-offs vary a lot but in principle it’s around 10 when we consider it is high, and below 10 it is low … so it’s a definition, well, that is quantitative but it is quite dirty and simplified, the way we calculate this, because why are we calculating this? Because we want to get a rough estimate of the number of neoantigens possibly recognized by the immune system … (Immuno-oncologist)

The TMB does not specify the neoantigens as such but assumes that these exist through a correlation: the higher the rate of somatic mutations in cancer cells, the higher their impact on the cells’ protein products, and hence the higher the possibility that some of these abnormal proteins could be recognized by the immune system as neoantigens. Currently, in clinical decision-making, a high TMB can push clinicians to consider the potential efficacy of a whole range of cancer immunotherapies. TMB is then considered as a biomarker for the administration of immune-checkpoint inhibitors, since these drugs can unleash the T cell response against neoantigens. Furthermore, a high TMB gets narrated as potentially predicting the response to ACT because it would mean that TILs extracted from the patient’s tumor may be highly specific to a mutational landscape of cancer cells enriched with neoantigens. However, the TMB is currently considered a ‘dirty’ measure by our informant because it is used without identifying a clear threshold and without completely understanding the mechanisms at stake. Little is known about the relationship between the mutation rate of the whole cancer genome and the regions that encode for antigen proteins (i.e., how many of these mutations have immunological effects). This is also problematic for clinicians as taking TMB into consideration restricts immunotherapies’ use to tumors with high TMB, such as lymphomas or melanoma, despite laboratory evidence that tumors with low TMB can also produce neoantigens (Bobisse et al., 2018).

The methods for investigating neoantigens documented above may be interpreted as a complement to the TMB’s capacity of roughly estimating cancer immunogenicity (Mattos-Arruda et al., 2020). For now, identifying each neoantigen is still in its infancy: It requires mobilizing platforms that are not yet connected to, or fully validated within (e.g. thourgh regulatory approval) the decisional space of the MTB, namely, immunopeptidomics and immune monitoring. Yet, this does not prevent clinician-researchers from integrating neoantigens into the thought processes, routines, and clinical gaze of MTBs, as in the case of early phase clinical trials. This happens through discussions around a patient’s TMB, which testify to a programmatic, yet unrealized at the time of our research, effort to bring concepts and tools of tumor immunology into clinical spaces where genomic technologies and knowledge are preeminent. In other words, even though the translational hybridization (i.e., the GMP-ification) of neoantigens is yet to come, these entities already partake in the bio-clinical gaze on cancer enacted in the MTB. Although unspecified, as in the case of the TMB, neoantigens are entities encouraging a sociotechnical process that connects independent translational infrastructures (e.g., autologous cells immunotherapy facilities with pre-existing genomic infrastructures in the NCRC) and expands the salience of immunogenomics in oncological care. Neoantigen-based ACTs shape a common way of thinking that spans across clinical and biological research settings. This process of hybridization—conditional to the GMP-ification of research infrastructures for antigen discovery and their implementation in the TMB—is far from complete. Yet, the work around neoantigens we observed in the NCRC appears to put the clinical research infrastructures of genomics and immunology on a converging trajectory.

## Discussion

In this article, we have explored the emergence of precision immuno-oncology. We have documented how current developments in cancer immunotherapy relate to the dominant approach enabled by genomics. Genomics is at the core of the thought styles, patterns, methods, and practices that make up so-called ‘precision oncology’. It is part of the concept, the machineries of knowing cancer, and the different institutional settings of expert practice of biomedical institutions. Put differently, it defines a ‘distinctive sociotechnical regime’ in oncology (Nelson et al., 2013, p. 407). Building upon this recognition, we have focused on the NCRC. There, we observed that neoantigens enable a major epistemic, technical, and organizational process that foregrounds a new ‘wave of enthusiasm’ surrounding immunology in the history of oncology (Löwy, 1996). We qualified the convergence of genomics and immunology in so-called ‘precision oncology’ as a hybridization process which touches upon their conceptual frameworks, technical/experimental infrastructures, and organizational configurations. Our reading underlines the conditions of possibility for change rather than the stabilization and inner workings of a successful tranformation of the dominant regime of genomic ‘precision’ in oncology. In a nutshell, hybridization emphasizes the open-endedness and incompleteness of these processes. While loosely structured convergences, connections, and agreements are in place, hybridization is far from delivering on its promise. Cancer immunotherapies, including ACTs, can thrive through the conceptualization of new hybrid biological entities (e.g. neoantigens). Yet, these entities can still be regarded as fully contiguous with either genomic or immunological views of cancer. Also, a shift in the understanding of ‘precision’ in oncology is conditional on the crafting of new ties between practical domains, experimental/technical infrastructures, and professional communities. And yet, these spaces are still far from coalescing into a new stable infrastructure of research and/or care, or a recognizable regime of biomedical practice.

Drawing on previous work in STS (Löwy, 1994; Rheinberger, 1997), we expand on hybridization as an analytical concept for analyses of change in the established sociotechnical regimes of biomedicine. We differentiate its analytical outlook in conceptualizing biomedical innovation from one offered by boundary objects. From this perspective, ‘loose concepts’ tied to boundary objects enhance collaborations between different epistemic communities (Löwy, 1992), which can lead to more robust concepts and stabilize a new subdiscipline (Löwy, 1994). While acknowledging this perspective, our use of hybridization underlines instead how convergence and stabilization, not just uncertainties and ambiguities, are functional to the unfolding of these collaborations. There exist, in other words, different concrete outcomes of these sociotechnical processes, which go beyond affording different actors opportunities to find common ground and work together prior to achieving consensus. In fact, we have highlighted different ways neoantigens allow actors to redefine how we know, experiment with, and treat cancer. At a theoretical level, our informants certainly experiment with neoantigens, while holding onto the boundary between immunological and genomic explanations of cancer. Yet, neoantigens also represent a hybrid concept in the making that, while unfinished, is fundamentally generative of shifts and articulations between different traditions and approaches to cancer. More than making the theoretical tenets of ‘precision’ thrive in ambiguity (as a boundary object would do), neoantigens set in motion the production of an explanation of cancer that gives both immunological and genomic ideas a crucial position in the operationalization of ‘precision’ in oncology. Similarly, at the level of experimental hybridization, the identification of neoantigens is a way to connect and demarcate scientists’ positioning in an experimental pipeline for the identification and validation of actionable cancer biomarkers. While the procedure for validating neoantigens is still uncertain, what is at stake is the convergence—not merely the co-existence—of different experimental systems, infrastructures of research, and epistemic communities. Finally, the same could be argued regarding the translational reach of this process. Beyond making the dwelling of clinical genomics and immunology in the oncology ward possible (Chiapperino et al., 2021), neoantigens set in motion the coordination and institutionalization of these competing platforms of clinical research at the NCRC. Enabled by a specific (yet unfinished) regulatory work of standardization and validation (i.e., GMP-ification), clinical oncologists narrate neoantigens as a coming therapeutic innovation (see Mattos-Arruda et al., 2020), if they do not already discuss them as entities worth clinical attention. Our analysis needs certainly further scrutiny, since it may be biased by our focus on one (leading) cancer research institute in immuno-oncology. Still, we argue that the stakes in neoantigens-based therapies no longer concern the porosity of a given boundary, a boundary that makes immunology attractive for researchers while still maintaining the borders between epistemic communities (see Löwy, 1996). Rather, neoantigens are at the core of a distinctive kind of epistemic work (i.e., hybridization) that sets on the trajectory of *producing* (without necessarily accomplishing) the compatibility of distinct traditions, theories, and configurations of research in oncology.

In characterizing our observations in terms of multiple forms of hybridization, we wish to highlight the heterogeneous junctions required to accomplish this goal. There are different and overlapping forms of hybridization at the NCRC: Different standards, distinct communities of practice, experimental approaches, and technologies have to blend together to produce a shift in the understanding of ‘precision’ in cancer research. Neoantigens can show that changing a dominant sociotechnical regime of biomedicine requires hybridization that affects the epistemic, technical, organizational, decisional, and regulatory underpinnings at its foundations. While these dimensions of oncology have all attracted substantive attention in STS (Bourret & Cambrosio, 2019; Kerr et al., 2019; Tempini & Leonelli, 2021), our analysis suggests that hybridities and demands for change often touch upon and straddle all these different dimensions. Neoantigens prompt a hybrid view of ‘precision’ that links the circuitry of culturing cells typical of immunologists (among others; cf. Landecker, 2007) with the technical possibilities of studying and understanding cancer through genomics, characterized by big data sequencing platforms (Tempini & Leonelli, 2021) and MTB (Bourret & Cambrosio, 2019). ‘Precision’ as enabled by the therapeutic actionability of neoantigens means restoring and enhancing an endogenous immune response against a tumor—hence it is the product of immunosurveillance and immune-editing. Yet, it also entails leveraging the biological singularities of cancer’s mutanome for therapeutic purposes. For this reason, the ‘precision patient’ at stake here is likely to be defined in terms of the genomic events and knowledge that produce cancer (Dam et al., 2022), all while being cared for through the practices and experimental infrastructures of immunology (Llewellyn, 2021; Swallow, 2024). Furthermore, these hybridities around ‘precision’ trouble the existing reputational and organizational hierarchies between epistemic regimes of innovation. Along with molecular biologists, immunologists hold a central position in the epistemic, experimental, and translational work required to develop more targeted cellular therapies. Thus, who gets to define ‘precision’ through neoantigens—and for whom—is a matter of challenging established jurisdictions that are fundamental to ‘precision oncology’ (cf. Bergeron et al., 2021). The concepts, tools, and practices of genomics in ‘precision oncology’ undeniably hold higher epistemic status and organizational prevalence at a global scale (Nelson et al., 2013, 2014). Yet, our fieldwork illustrates how the translational research involving neoantigens challenges this established technical, cognitive and clinical hierarchy through nascent situated synergies (see Crabu, 2018). More than a conflictual reconfiguration of power relations between different traditions of oncological research, the NCRC witnesses multiple hybridization processes that demand a novel equilibrium grounded in increasingly stable networks of collaboration and infrastructures of research. Finally, beyond our focus on epistemic change, the sociotechnical processes we describe are attracting major scientific and economic investments—notably, in the ‘emerging tissue economies’ related to autologous cell therapies (including ACTs, therapeutic vaccines, or CAR-T; Llewellyn, 2021). Because it bridges two therapeutic traditions and corresponding infrastructures, ‘precision immuno-oncology’ is likely to hold the highest professional, public, and economic expectations of the bubbling sector of precision oncology (Erikainen & Chan, 2019; Kerr et al., 2019). In this sense, ‘precision immuno-oncology’ extends—more than it challenges—the ‘scientific ideology’ of ‘precision medicine’ (Löwy, 2022), which envisions erasing clinical and biological boundaries to bring direct benefit to patients through tailored treatments at both genomic and immunological levels. While we have shed light on concrete changes at the conceptual, experimental, and translational levels, we have also shown that the ideal of convergence at stake is still fragile and unstable in practice, especially at clinical levels where the immunotherapeutic (i.e., manufacturing facilities and dedicated hospitalization units) and genomic (i.e., the MTB) bio-clinical infrastructures remain separate. Furthermore, it remains to be seen whether this ideal of translational research may once again accelerate access to innovative treatments typical of ‘precision oncology’ but not translate into clinical benefits and equitable care (Kerr, 2021; Prasad, 2016).

Our focus on hybridization emphasizes the interplay of dimensions that sets in motion change in the established sociotechnical regimes of science. Our case study has therefore wider stakes than the boundary work around an epistemic entity (Star, 2010), and/or the stabilization of innovations into biomedical platforms (Keating & Cambrosio, 2003). Without the need to reaffirm the importance of these concepts, our analysis of neoantigen-based ACT highlights that innovation is also the result of open, uncertain, and multidimensional alignments that are generative of social and epistemic change. While hybridization has been described as an open-ended, experimental process, prone to bifurcation and unexpected amalgamations (Rheinberger, 1997), our article is a reminder that it may be so beyond epistemic practices. Hybridization may not simply be the generator of ‘unprecedented, surprising [experimental] results’ (Rheinberger, 1997, S247). Rather, it shows that change happens through an active articulation that straddles the epistemic and the social (Löwy, 1994): Immunology and genomics have only initiated (as illustrated by the case of the NCRC) their convergence by making hybrid configurations of biomedical practices. Our take on hybridization emphasizes the unfinished and unstable character of these processes; it is, after all, a hybrid*ization* in the making that we observed, whose (epistemic, organizational, regulatory, socioeconomic, etc.) outcomes are fundamentally to be written. The high degree of singularization and technicity of neoantigen discovery, T cell isolation and production—costly and time-consuming work—may limit the translation of ACT to wider clinical uses. In fact, the challenge of making neoantigens more ‘actionable’ in the clinic still stands at the moment (Mattos-Arruda et al., 2020). Neoantigens also compete in the translational pipeline with other biological and bio-clinical entities producing (*prima facie*) similar hybridizations, such as those related to engineering T cells (CAR-T therapy), therapeutic vaccines, or drugs targeting the tumor microenvironment. In view of this, our argument should not be mistaken for an assessment of their promissory value as therapeutic. Rather, neoantigens may only be an exemplary case to dissect the multiple concepts, technologies, platforms, and values that are conditional to changing the sociotechnical regimes of science and innovation, for better and for worse.

## Notes

1. In the introduction and discussion, we use the word ‘precision’ within scare quotes. This is meant to underline that the way sociotechnical regimes are spoken about is not neutral. While in the rest of the article we drop the scare quotes for the sake of readability, we alert the reader about the value baggage manufactured through a terminology of ‘precision’ associated to biomedical innovation (Erikainen & Chan 2019).
